# Highly Efficient Transfer of Chromosomes to a Broad Range of Target Cells Using Chinese Hamster Ovary Cells Expressing Murine Leukemia Virus-Derived Envelope Proteins

**DOI:** 10.1371/journal.pone.0157187

**Published:** 2016-06-07

**Authors:** Teruhiko Suzuki, Yasuhiro Kazuki, Mitsuo Oshimura, Takahiko Hara

**Affiliations:** 1 Stem Cell Project, Tokyo Metropolitan Institute of Medical Science, Tokyo, Japan; 2 Department of Biomedical Science, Institute of Regenerative Medicine and Biofunction, Graduate School of Medical Science, Tottori University, Tottori, Japan; 3 Chromosome Engineering Research Center, Tottori University, Tottori, Japan; Imperial College London, UNITED KINGDOM

## Abstract

Microcell-mediated chromosome transfer (MMCT) is an essential step for introducing chromosomes from donor cells to recipient cells. MMCT allows not only for genetic/epigenetic analysis of specific chromosomes, but also for utilization of human and mouse artificial chromosomes (HACs/MACs) as gene delivery vectors. Although the scientific demand for genome scale analyses is increasing, the poor transfer efficiency of the current method has hampered the application of chromosome engineering technology. Here, we developed a highly efficient chromosome transfer method, called retro-MMCT, which is based on Chinese hamster ovary cells expressing envelope proteins derived from ecotropic or amphotropic murine leukemia viruses. Using this method, we transferred MACs to NIH3T3 cells with 26.5 times greater efficiency than that obtained using the conventional MMCT method. Retro-MMCT was applicable to a variety of recipient cells, including embryonic stem cells. Moreover, retro-MMCT enabled efficient transfer of MAC to recipient cells derived from humans, monkeys, mice, rats, and rabbits. These results demonstrate the utility of retro-MMCT for the efficient transfer of chromosomes to various types of target cell.

## Introduction

Human artificial chromosomes (HACs) and mouse artificial chromosomes (MACs) are unique gene delivery vectors that behave like natural chromosomes in mammalian host cells [1, 2]. Since HACs/MACs are capable of holding megabase-sized DNA inserts [3, 4], they have been used to generate unique animal models such as Cyp-humanized mice and human antibody-producing mice or calves [5–7]. In addition, HACs/MACs are useful for introducing multiple gene expression units into target cells [8–10].

Chromosomes, including HACs/MACs, can be transferred from donor cells to recipient cells by microcell-mediated chromosome transfer (MMCT) [11]. The first step in the process is to treat donor cells with colcemid to induce micronuclei, each of which contains one or a few chromosomes. The cells are then treated with cytochalasin B to disrupt the cytoskeleton and enable enucleation of micronuclei. The resulting microcells are fused with target cells using polyethylene glycol (PEG), and the chromosomes are transferred. Although this is an essential step, its inefficiency, laboriousness, and cytotoxic effects of PEG have hampered the widespread application of the chromosome engineering technology. Alternatives to PEG-mediated MMCT (PEG-MMCT), such as the introduction of purified HACs/MACs using commercially available transfection reagents [12] or micronucleated whole cell fusion, have also been developed [13, 14]. Although there are some advantages to these methods, the efficiency of the former is comparable with that of PEG-MMCT and the latter seems to cause aneuploidy in target cells. Recently reported new chromosome transfer method utilizing measles virus envelope proteins or measles virus envelope proteins fused to a single chain antibody substantially improves the overall efficiency of MMCT [15, 16]. However, the method is only applicable to human cells due to the host range of the measles virus.

Murine leukemia viruses (MLVs) are retroviruses that can be divided into six subclasses based on their host range [17, 18]. Of these, ecotropic and amphotropic MLVs are the most well characterized types. Ecotropic MLV recognizes only mouse and rat cells by binding to cationic amino acid transporter-1 (Cat-1), whereas amphotropic MLV infects a wide range of mammalian cells (including mouse, rat, rabbit, monkey, and human) by binding to the sodium-dependent phosphate transporter, Pit-2. Both receptors are ubiquitously expressed membrane proteins conserved in mammals. The envelope protein (Env) of MLV consists of surface (SU) and transmembrane (TM) components, both of which are derived from a single precursor protein encoded by the *env* gene. The SU component is responsible for recognizing the receptor protein, while TM mediates membrane fusion. The R-peptide, an intracellular domain within the TM component, inhibits Env-mediated membrane fusion, presumably to prevent inter-host cell fusion [19]. During maturation of the virion, the R-peptide is cleaved off by a viral protease to make Env fusion-competent.

Chinese hamster ovary (CHO) and mouse fibroblast-derived A9 cells are the most popular chromosome donor cells because of their highly efficient generation of microcells. Importantly, CHO cells are completely resistant to infection by ecotropic and amphotropic MLVs [19]. Based on these properties, we decided to utilize the R-peptide-deleted Env protein of MLVs as the fusogen for donor CHO-derived microcells to develop a highly efficient MMCT method.

## Results

### Development of the retro-MMCT method

**Fig 1:**
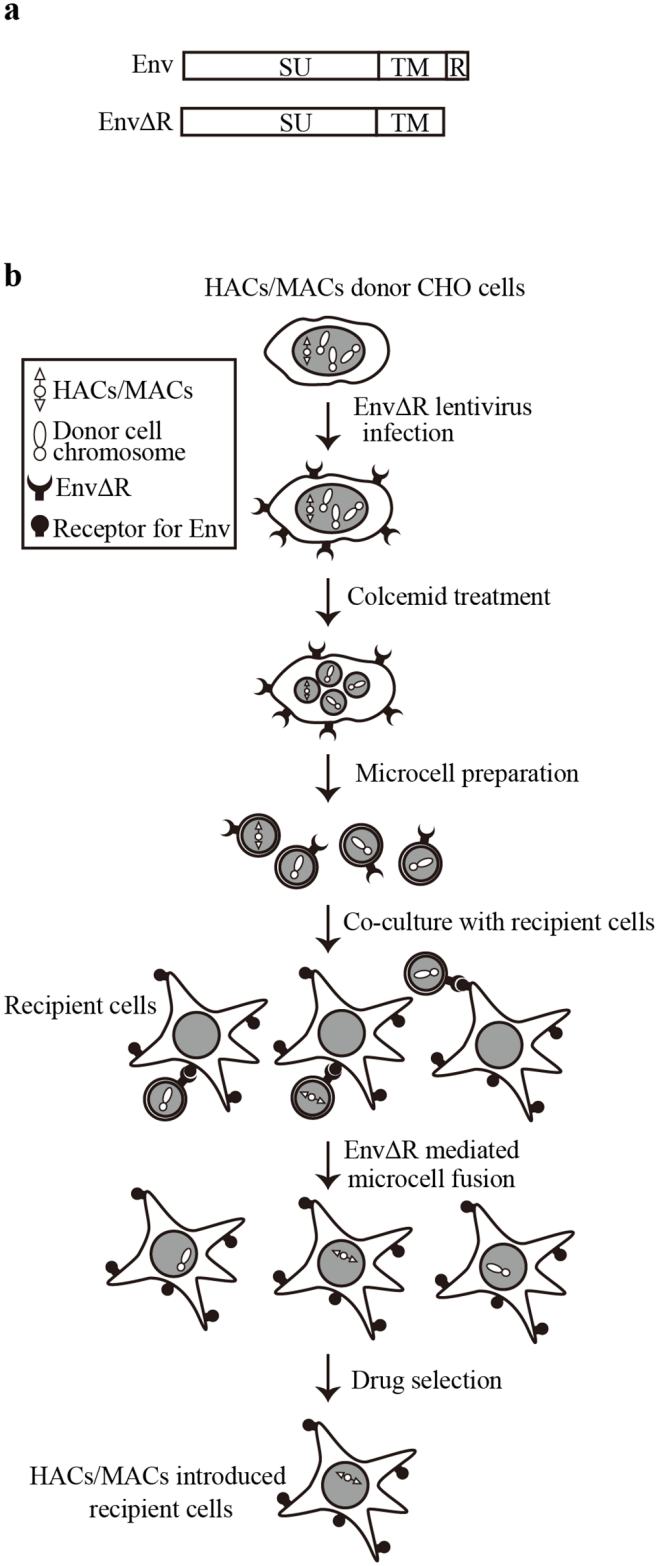
Overview of the retro-MMCT method. (a) Structure of Env and EnvΔR. SU, SU component; TM, TM component; R, R-peptide. (b) Schematic representation of retro-MMCT-mediated HACs/MACs transfer to recipient cells.

**Fig 2:**
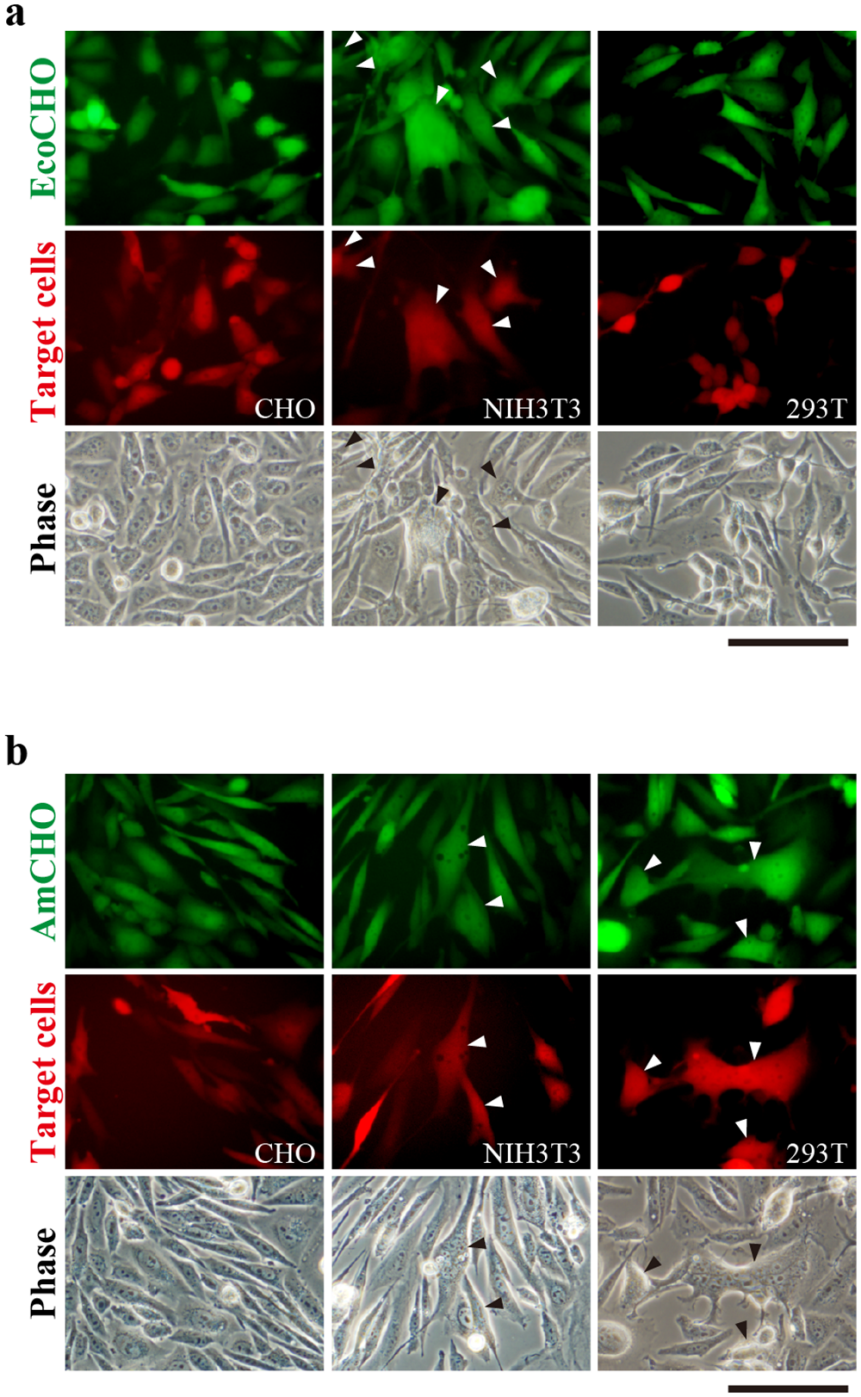
Cell fusion of EnvΔR-expressing CHO cells. Lentiviral vectors encoding ecotropic (a) or amphotropic (b) EnvΔR-IRES-EGFP were used to infect CHO cells (green, upper panels), which were then co-cultured with CHO, NIH3T3, or 293T cells labeled with TdTomato (red, middle panels). Arrowheads indicate EGFP^+^TdTomato^+^-fused cells. EcoCHO and AmCHO represent CHO cells transduced with ecotropic and amphotropic EnvΔR-IRES-EGFP, respectively. Phase contrast images are shown in the lower panels. Scale bars, 100 μm.

**Fig 3:**
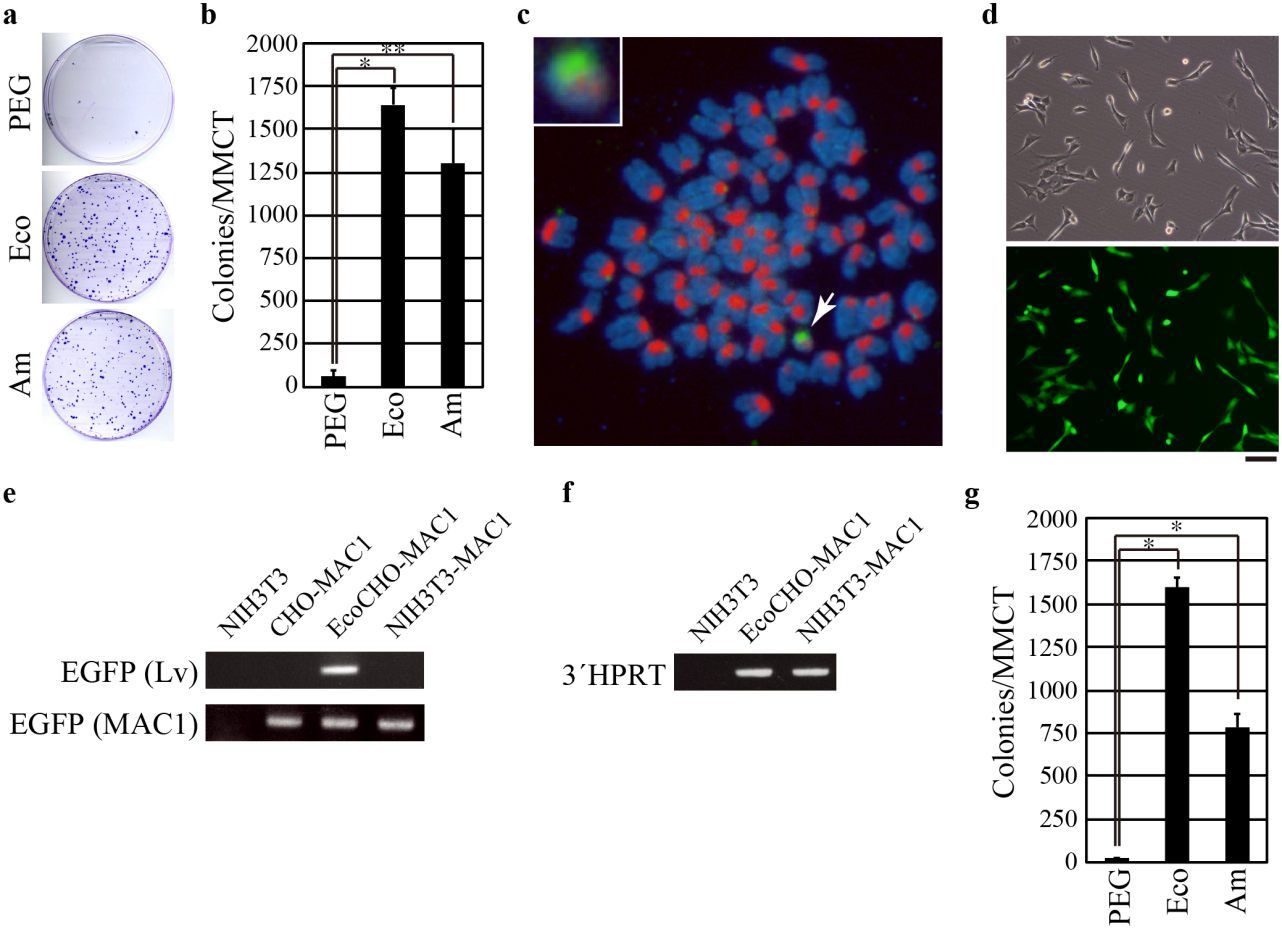
Transfer of MAC1 to NIH3T3 cells. (a) MAC1 was introduced into NIH3T3 cells using PEG-MMCT (PEG), Eco-MMCT (Eco), or Am-MMCT (Am). MAC1-transferred NIH3T3 cells were selected with G418, and colonies were stained with crystal violet. Representative images of the plates are shown. (b) Comparison of total colony numbers generated using the three MMCT methods. (c) FISH analysis of a MAC1-transferred NIH3T3 clone obtained by Eco-MMCT. Chromosomes of mouse origin were detected using a mouse major satellite probe (red). MAC1 (arrow) was identified with a Neo gene probe (green). The sample was counterstained with DAPI to visualize chromosomes (blue). Inset: high magnification image of MAC1. (d) Phase (upper panel) or fluorescent (lower panel) images of MAC1-transferred NIH3T3 clone. Scale bar, 100 μm. (e) Genomic PCR using primer sets specific for EGFP expression cassettes of lentivirus vector (EGFP (Lv)) or MAC1 (EGFP (MAC1)). Genomic DNA prepared from indicated cells was used as template. EcoCHO-MAC1, EcoCHO cells carrying MAC1; NIH3T3-MAC1, MAC1-transferred NIH3T3 cells obtained by Eco-MMCT. (f) Genomic PCR using a primer set for 3´HPRT cassette of MAC1. (g) Efficiency of retro-MMCT using cryopreserved microcells. MAC1 was introduced to NIH3T3 cells using cryopreserved microcells and all three MMCT methods. Each value represents the mean ± SD (*n* = 3; *P < 0.005; **P < 0.01).

**Fig 4:**
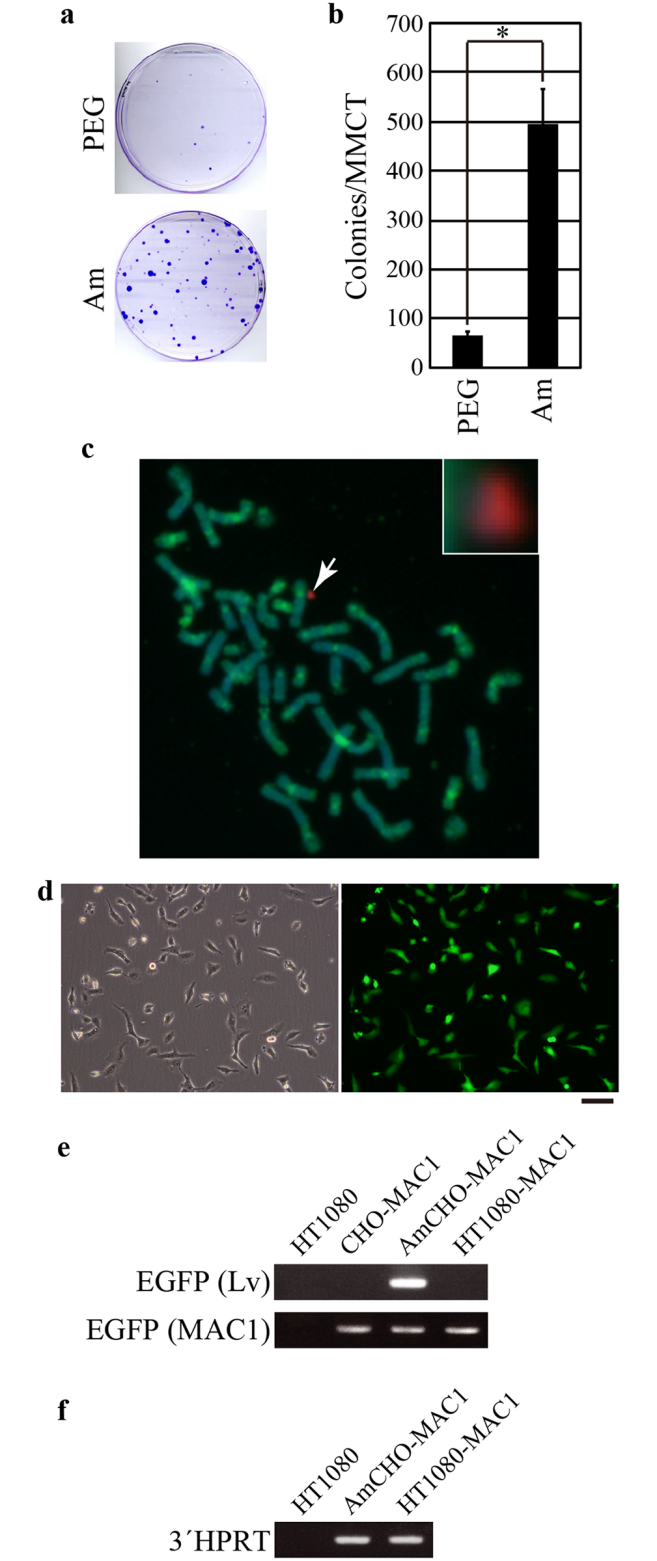
Transfer of MAC1 to HT1080 cells. (a) MAC1 was introduced into HT1080 cells using PEG-MMCT (PEG) or Am-MMCT (Am). MAC1-transferred G418-resistant HT1080 clones were stained with crystal violet. Representative images of the plates are shown. (b) Comparison of total colony numbers generated by the PEG-MMCT and Am-MMCT methods. Each value represents the mean ± SD (*n* = 3; *P < 0.01). (c) FISH analysis of a MAC1-transferred HT1080 clone obtained by Am-MMCT. MAC1 (arrow) was detected with a mouse major satellite DNA probe (red). Human chromosomes were detected using a human Cot-1 probe (Green). The sample was counterstained with DAPI to visualize chromosomes (blue). Inset: high magnification image of MAC1. (d) Phase (left panel) or fluorescent (right panel) images of MAC1-transferred HT1080 clone. Scale bar, 100 μm. (e) Genomic PCR using primer sets specific for EGFP expression cassettes of lentivirus vector (EGFP (Lv)) or MAC1 (EGFP (MAC1)). Genomic DNA prepared from indicated cells was used as template. AmCHO-MAC1, AmCHO cells carrying MAC1; HT1080-MAC1, MAC1-transferred HT1080 cells obtained by Am-MMCT. (f) Genomic PCR using a primer set for 3´HPRT cassette of MAC1.

We designed a new MMCT method, retro-MMCT (see Fig 1 for overview). We used CHO cells carrying MAC1 (CHO-MAC1), which harbors a neomycin-resistance gene, as chromosome donor cells for the proof-of-principle experiments [20]. Since deletion of the R-peptide makes Env fusion-competent [19], we used R-peptide-deleted Env (EnvΔR) from ecotropic or amphotropic MLVs to induce fusion between microcells and recipient cells (Fig 1a). EnvΔR expression units were introduced into CHO-MAC1 cells using VSVG-pseudotyped lentiviral vectors. Stable expression of ecotropic and amphotropic EnvΔR did not cause inter-donor cell fusion because CHO cells lack receptors for ecotropic and amphotropic Env (see Fig 2a and 2b, left columns). Since MAC1 is far smaller than the donor cell chromosomes, the lentiviruses are expected to integrate into the donor cell genome rather than MAC1 (see Figs 3e and 4e). Even if EnvΔR expression units integrate into MAC1, transfer of MAC1 kills recipient cells by inducing syncytium formation. Thus, MAC1 can be segregated from EnvΔR expression cassettes during retro-MMCT. Microcells prepared from CHO-MAC1 donor cells present EnvΔR on the surface, thereby inducing fusion with target cells. MAC1-introduced G418-resistant cells were obtained by simple co-culture of CHO-MAC1-derived microcells with target cells (see below).

### Cell fusion capacity of EnvΔR-transduced CHO cells

To determine whether stable expression of EnvΔR in CHO cells mediates fusion with MLV-permissive cells but not CHO cells, we co-cultured CHO cells expressing ecotropic EnvΔR-IRES-EGFP or amphotropic EnvΔR-IRES-EGFP with TdTomato-expressing CHO, mouse fibroblast-derived NIH3T3, or human fetal kidney-derived 293T cells. Neither ecotropic EnvΔR- (Fig 2a) nor amphotropic EnvΔR- (Fig 2b) expressing CHO cells fused with CHO cells, whereas both were capable of fusing with NIH3T3 cells. Amphotropic EnvΔR, but not ecotropic EnvΔR, also mediated fusion with 293T cells. These results indicate that cell fusion of EnvΔR-expressing CHO cells depends on the tropism of the envelope proteins.

### Efficiency of chromosome transfer to NIH3T3 cells by retro-MMCT

We next examined whether microcells prepared from EnvΔR-transduced CHO-MAC1 cells efficiently transfer MAC1 to target cells. Microcells were prepared from CHO-MAC1 cells expressing ecotropic EnvΔR or amphotropic EnvΔR and then incubated with NIH3T3 cells. MAC1-transferred NIH3T3 clones were selected with G418 and then counted. Retro-MMCT using ecotropic EnvΔR (Eco-MMCT) or amphotropic EnvΔR (Am-MMCT) generated 26.5 and 20.9 times more colonies, respectively, than the conventional PEG-MMCT method (Fig 3a and 3b). We next conducted fluorescence *in situ* hybridization (FISH) analysis to confirm that MAC1 was correctly introduced into recipient NIH3T3 cells (Fig 3c, arrow). All chromosomes were positive when tested using a mouse major satellite probe, indicating that chromosomes from CHO cells were not introduced to the recipient cells together with MAC1. To validate the integrity of MAC1, we confirmed the expression of EGFP, which is encoded by MAC1 (Fig 3d) [20]. Genomic PCR ensured that the EGFP cassette of MAC1, but not the EnvΔR-IRES-EGFP unit of lentivirus vector was responsible for the fluorescence (Fig 3e). This result represented that the lentivirus vectors were not integrated in MAC1 as expected. MAC1 of NIH3T3 clone also retained 3´HPRT cassette, which is an essential component of MAC1 for the gene loading (Fig 3f). These results demonstrated that truncation of MAC1 did not occur after retro-MMCT-mediated chromosome transfer.

A previous report shows that microcells can be frozen and stored at -80°C [21]. Therefore, we asked whether microcells for retro-MMCT could be cryopreserved. We found that the microcells used for retro-MMCT retained high chromosome transfer efficiency even after freezing at -80°C (Fig 3d). These results show that retro-MMCT is a highly efficient and convenient method for chromosome transfer.

### Efficiency of chromosome transfer to various mammalian cells by retro-MMCT

Since amphotropic MLV infects various mammalian cells other than mouse cells, we next used Am-MMCT to transfer MAC1 into human HT1080 fibrosarcoma cells. We found that Am-MMCT was 7.6 times more efficient than PEG-MMCT (Fig 4a and 4b). FISH analysis verified the presence of MAC1 in recipient HT1080 cells (Fig 4c, arrow). Furthermore, all the chromosomes except MAC1 were positive for human Cot-1 probe, which specifically detects human chromosomes. This result proved that chromosomes of donor cell origin are not introduced together with MAC1. To validate the integrity of MAC1 in the recipient HT1080 cells, we confirmed the expression of EGFP (Fig 4d). Genomic PCR ensured that the EGFP cassette of MAC1, but not the EnvΔR-IRES-EGFP unit of lentivirus vector was responsible for the expression of EGFP (Fig 4e). MAC1 of HT1080 clone also retained 3´HPRT cassette (Fig 4f). These results showed the integrity of MAC1 in the HT1080 clone.

Finally, we applied retro-MMCT to various human, monkey, rabbit, mouse, and rat cell lines, and found that retro-MMCT was effective in all cases (Table 1). It is noteworthy that retro-MMCT enabled efficient transfer of MAC1 to various type of cells including ES cells. Taken together, these results demonstrate the utility of retro-MMCT for the transfer of chromosomes to various target cells.

**Table 1:** Comparison of MMCT efficiency.

Target cell line	Species	Cell origin	Colonies/MMCT		
			PEG-MMCT	Eco-MMCT	Am-MMCT
MCF7	Human	Breast adenocarcinoma	30	NA	174 (5.8)
HeLa	Human	Cervix carcinoma	3	NA	36 (12.0)
Cos7	African green monkey	Kidney	54	NA	453 (8.4)
Sirc	Rabbit	Cornea	3	NA	120 (40.0)
E14Tg2a	Mouse	Embryonic stem cell	5	180 (36.0)	290 (58.0)
NB2a	Mouse	Neuroblastoma	21	768 (36.6)	681 (32.4)
CMT-93	Mouse	Rectum	51	66 (1.3)	141 (2.8)
Rat-1	Rat	Fibroblast	15	660 (44.0)	195 (13.0)

Total colony numbers obtained by each MMCT method are shown. The fold increase against PEG-MMCT is shown in the parentheses. NA; data not available.

## Discussion

The low efficiency of the conventional MMCT method is an obstacle to the advancement of chromosome engineering technology. Here, we overcame this limitation by developing the retro-MMCT method, which enables the introduction of MAC1 to NIH3T3 cells at a frequency of 5.5 × 10^−3^ colonies per recipient cell without any manipulation of the recipient cells. Although the efficiency depends on the experimental settings, the chromosome transfer efficiency is higher than any other methods including HVJ-E mediated MMCT to the best of our knowledge [22]. It is worth noting that the high transduction efficiency was obtained with only one T25 flask of donor cells. In the case of the conventional PEG-MMCT method, we normally prepare microcells from 6 to 24 flasks of donor cells. Thus, the retro-MMCT method significantly reduces both the duration and cost of chromosome transfer experiments. Importantly, retro-MMCT method utilizes only envelope proteins of MLVs expressed on the cell-surface and does not require packaging as virus vectors. Thus, there is no limitation of the size of insert DNA in HACs/MACs when retro-MMCT method is used to transfer chromosomes. This method not only mediates efficient microcell-to-target cell fusion, but also eliminates the toxicity associated with PEG treatment, which contributes to the low efficiency of the conventional method. The efficiency of Eco-MMCT was better than that of Am-MMCT in some type of cells, but not in other cell lines (Fig 3 and Table 1). This may be due to expression levels of the receptor proteins Cat-1 and Pit-2. Thus, while both retro-MMCT methods showed better results than PEG-MMCT, evaluation is needed to determine which method (Eco-MMCT and Am-MMCT) gives the highest chromosome-transfer efficiency to specific target cells.

We used CHO cells as donor cells because they are resistant to infection by ecotropic and amphotropic MLVs. Since the EnvΔRs induce inter-host cell fusion of MLV-permissive cells, mouse-derived A9 cells, an alternative cell line used as a chromosome donor cell, are not appropriate chromosome-donor cells for use in retro-MMCT. However, it was mentioned that L cell derivatives, including A9 cells, were resistant to infection by ecotropic MLV [23]. Thus, Eco-MMCT may be applicable to A9 donor cells. Moreover, a previous study reported that both *Slc7a1* (a gene encoding an ecotropic MLV receptor, Cat-1) and *Slc20A2* (a gene encoding an amphotropic MLV receptor, Pit-2) knockout mice are viable, at least until the perinatal stage, suggesting that these genes are not necessary for culture *in vitro* [24, 25]. Therefore, disrupting these genes using CRISPR/Cas9 would enable transfer of chromosomes from A9 donor cells using the retro-MMCT method.

Since the efficiency of retro-MMCT is extremely high compared to any other methods, it may enable introduction of HACs/MACs into spermatogonial stem cells, hematopoietic stem cells and human iPS cells that have been considered practically impossible to transfer HACs/MACs. Thus, retro-MMCT method would open a way for rapid generation of humanized mouse strains and new disease models. The retro-MMCT method might also be applicable to *in vivo* chromosome transfer. Conventional PEG-MMCT requires PEG treatment to achieve microcell fusion; therefore, the method cannot be used *in vivo*. By contrast, a simple injection of microcells into recipients would be sufficient for the retro-MMCT method. Although this technique would require the isolation of microcells containing only HACs/MACs, the retro-MMCT method could be used to deliver HACs/MACs carrying huge DNA inserts, such as the DMD gene locus or multiple gene expression units, for gene therapy of Duchenne muscular dystrophy and *in vivo* direct reprogramming, respectively.

## Materials and Methods

### Cell culture

CHO cells (HPRT-deficient derivative) (JCRB Cell Bank) were cultured in F12 medium supplemented with 10% FCS. NB2a (RIKEN BioResource Center) was cultured in RPMI 1640 supplemented with 10% FCS and 100 μM 2-mercaptoethanol. E14Tg2a (a kind gift from Dr. H. Niwa, RIKEN, Japan) was cultured as described previously [26]. All other cell lines were cultured in DMEM supplemented with 10% FCS.

### Vector construction

Moloney MLV-derived ecotropic envelope and 4070A MLV-derived amphotropic envelope genes were cloned using genomic DNA of PLAT-E and PLAT-A packaging cells (kind gifts from Dr. T. Kitamura, Tokyo University, Japan), respectively, as templates [27]. The envelope genes lacking the R-peptide region were cloned into pWPI (a kind gift from Dr. D. Trono, EPFL, Switzerland) to prepare the lentivirus vector. The EGFP gene downstream of the IRES element in pWPI was replaced with TdTomato to label cells with TdTomato. Lentiviruses were prepared as described previously [26]. Polyethylenimine “Max” (Polysciences) was used for transient expression to prepare lentivirus.

### Microcell-mediated chromosome transfer

MMCT was performed as described previously, with some modifications [6]. For retro-MMCT, MAC1 donor CHO cells were infected with VSV-G pseudotyped lentiviral vectors (MOI = 3) encoding EnvΔR. To analyze the efficiency of MMCT, 2.3 × 10^6^ MAC1 donor CHO cells were seeded into one T25 flask. On the next day, the medium was changed to colcemid medium (Ham’s F12 medium containing 20% FCS, penicillin/streptomycin, and 0.1 μg/ml demecolcine (Wako)). Two days after treatment, the medium was again replaced with fresh colcemid medium. Microcells were prepared on the next day. The microcells for retro-MMCT were cultured with 3 × 10^5^ recipient cells, which had been inoculated into a well of a 6 well plate 1 day before. The same number of recipient cells was used for PEG-MMCT. One third of the recipient cells were replated into a 10 cm dish 1 day after MMCT. G418 was added 48 hours after MMCT, and cells were cultured for 7 to 10 days to select those harboring MAC1. The G418-resistant clones were stained with 0.05% crystal violet to analyze the efficiency of MMCT. The number of colonies per MMCT was calculated by multiplying the number of clones in the 10 cm dish by three. To examine the efficiency of chromosome transfer using cryopreserved microcells, microcells prepared as above were stored at -80°C using Cellbanker 1 (ZENOAQ). Student's *t* test was performed to evaluate statistical significance.

### FISH analysis

FISH analysis was performed as described previously [8]. In brief, a digoxigenin-labeled probe specific for a mouse major satellite sequence and a biotin-labeled probe specific for the neomycin-resistance gene or human Cot-1 DNA (ThermoFisher Scientific) were prepared using Nick Translation Mix (Roche). The TSA Biotin Kit (PerkinElmer) was used to enhance the signal generated by the neomycin-resistance gene probe. Samples were counterstained with 4´,6-diamidino-2-phenylindole (DAPI) to visualize genomic DNA.

### Genomic PCR

Genomic DNA prepared from cultured cells was subjected for PCR using OneTaq DNA Polymerase (New England Biolabs). Primers used to detect EGFP (Lv), EGFP (MAC1) and 3´HPRT were as follows: EGFP (Lv) (5´-TGCGGCCAAAAGCCACGTGTATAAG-3´ and 5´-CTCGCCGGACACGCTGAACTTGT-3´); EGFP (MAC1) (5´-CTGCTGCCCGACAACCACTA-3´ and 5´-CCAGAAGTCAGATGCTCAAGGGGC-3´); 3´HPRT (5´-CAGTGGCACAATCACAGTTCACTCC-3´ and 5´-TCGAGCAAGACGTTCAGTCCTACAG-3´).
